# Proteomic Analysis of Mouse Kidney Tissue Associates Peroxisomal Dysfunction with Early Diabetic Kidney Disease

**DOI:** 10.3390/biomedicines10020216

**Published:** 2022-01-20

**Authors:** Aggeliki Tserga, Despoina Pouloudi, Jean Sébastien Saulnier-Blache, Rafael Stroggilos, Irene Theochari, Harikleia Gakiopoulou, Harald Mischak, Jerome Zoidakis, Joost Peter Schanstra, Antonia Vlahou, Manousos Makridakis

**Affiliations:** 1 Department of Biotechnology, Biomedical Research Foundation, Academy of Athens, Soranou Efessiou 4, 11527 Athens, Greece; atserga@bioacademy.gr (A.T.); rstrog@bioacademy.gr (R.S.); izoidakis@bioacademy.gr (J.Z.); 2 First Department of Pathology, School of Medicine, National and Kapodistrian University of Athens, 11527 Athens, Greece; dpouloudi@med.uoa.gr (D.P.); theoirene@med.uoa.gr (I.T.); chgakiop@med.uoa.gr (H.G.); 3 Institut National de la Santé et de la Recherche Médicale (INSERM), U1297, Institute of Cardiovascular and Metabolic Disease, 31432 Toulouse, France; jean-sebastien.saulnier-blache@inserm.fr; 4 Université Toulouse III Paul-Sabatier, 31062 Toulouse, France; 5 Mosaiques Diagnostics GmbH, 30659 Hannover, Germany; mischak@mosaiques.de

**Keywords:** diabetes, diabetic kidney disease, proteomics, kidney, LC–MS/MS, biomarker, peroxisome, glomeruli

## Abstract

Background: The absence of efficient inhibitors for diabetic kidney disease (DKD) progression reflects the gaps in our understanding of DKD molecular pathogenesis. Methods: A comprehensive proteomic analysis was performed on the glomeruli and kidney cortex of diabetic mice with the subsequent validation of findings in human biopsies and omics datasets, aiming to better understand the underlying molecular biology of early DKD development and progression. Results: LC–MS/MS was employed to analyze the kidney proteome of 2 DKD models: Ins2Akita (early and late DKD) and db/db mice (late DKD). The abundance of detected proteins was defined. Pathway analysis of differentially expressed proteins in the early and late DKD versus the respective controls predicted dysregulation in DKD hallmarks (peroxisomal lipid metabolism and β-oxidation), supporting the functional relevance of the findings. Comparing the observed protein changes in early and late DKD, the consistent upregulation of 21 and downregulation of 18 proteins was detected. Among these were downregulated peroxisomal and upregulated mitochondrial proteins. Tissue sections from 16 DKD patients were analyzed by IHC confirming our results. Conclusion: Our study shows an extensive differential expression of peroxisomal proteins in the early stages of DKD that persists regardless of the disease severity, providing new perspectives and potential markers of diabetic kidney dysfunction.

## 1. Introduction

According to the 2019 Atlas of the International Diabetes Federation, approximately 463.0 million adults have type 1 (T1D) or type 2 diabetes (T2D) [1,2] Significant highest prevalence rates are observed in youth-onset T2DM mainly due to increased childhood obesity [3]. Diabetic patients are at high risk of developing DKD, cardiovascular disease (CVD), neuropathy and retinopathy [1].

The prevalence of DKD is increasing and is associated with a heavy societal and financial burden [4] and therapeutic inertia, creating a major problem in DKD and diabetes treatment [5]. DKD is characterized by altered glomerular filtration and proteinuria resulting in up to 50% of the patients with end stage kidney disease (ESKD) due to DKD [6,7]. Currently, early prevention and management of DKD remain limited. To date, the diagnosis of DKD is based on clinical features; however, the combination with a kidney biopsy is significant [8]. DKD is characterized by kidney ultra-structural and morphological alterations such as mesangial expansion, nodular glomerular sclerosis, glomerular basement membrane (GBM) thickening and tubulointerstitial fibrosis [9]. Kidney fibrosis is the downstream effect of activation due to hyperglycemia of several signaling pathways such as TGF-β [10], JAK/STAT [11] and Notch [12] leading to oxidative stress. Endothelial glucocorticoid receptors (GRs) are crucial regulators of DKD fibrosis as demonstrated in a recent study where the loss of endothelial GRs in diabetic mice resulted in an increased Wnt/β-catenin pathway and decreased FAO and increased fibrinogenesis [13]. However, endothelial SIRT3, a mitochondrial sirtuin, blocks kidney fibrosis through TGF-β/smad pathway regulation [14]. Glomerular injury characterizes the early stages of DKD; thus, glomeruli are significant targets to investigate the molecular mechanisms of early DKD pathogenesis [15].

Several biological processes relevant to DKD have been studied, such as mitochondrial dysfunction [16], reactive oxygen species (ROS) production [17], NADPH oxidase (NOX) activity [18], podocyte apoptosis and autophagy [19] leading to glomerular injury [20]. Oxidative stress has been highlighted as a significant contributor to DKD and progression to ESKD [21,22,23], being directly linked to podocyte damage, proteinuria, and tubulointerstitial fibrosis [23]. Oxidative stress is triggered from changes in kidney lipid metabolism [24] with kidney lipotoxicity and lipid accumulation being considered pathological hallmarks of DKD [24,25]. Glomerular lipid accumulation could lead to podocyte death and insulin resistance [24,25]. Despite this accumulated knowledge, the absence of efficient inhibitors for the progressive distortion of kidney structure and function reflects the gaps in our understanding of DKD pathogenesis [26]. The most promising treatment of DKD currently is inhibition of sodium–glucose transporter 2 (SGLT2). SGLT2 inhibitors, such as dapagliflozin [27], canagliflozin [28] and empagliflozin [29], were initially developed to lower blood glucose concentrations but also showed very favorable protective effects in DKD apparently independent of the blood glucose lowering effect. Other promising hypoglycemic drugs than SGLT2i are GLP-l receptor agonists [18,30] and DPP-4 inhibitors [31] used in animal studies and clinical trials improving blood glucose control and reducing albuminuria [32]. Another promising drug for DKD are the mineralocorticoid receptor antagonists (MRAs) diminishing the activation of kidney inflammation and fibrosis [33]. Newly developed drugs targeting JAk/STAT, TGFb and PKC pathways and ROCK inhibitors show promising results in animal studies lowering albuminuria and/or kidney inflammation and fibrosis podocyte injury [32].

Many mouse and rat models of T1D and T2D have been established in order to dissect diabetic and DKD pathogenesis [34]. Several DKD studies use Ins2Akita mice as models of T1D since these mice have glomerular basement membrane thickening, increased albumin excretion, glomerulosclerosis and interstitial fibrosis, which mimic human DKD [35]. Hyperglycemia in Ins2Akita mice is thought to induce oxidative stress, resulting in kidney injury [36]. Additionally, the db/db mouse model is frequently used as a model of human T2D due to susceptibility to obesity, insulin resistance and T2D resulting from leptin deficiency and the development of progressive histological lesions in their kidneys [34].

To shed more light onto the molecular mechanisms of early DKD pathogenesis and progression, our study targeted the comprehensive molecular characterization of kidney tissue compartments at different developmental time points from the widely used Ins2Akita model of DKD with the subsequent validation of findings in the db/db model and in human kidney biopsies. We performed a high-resolution, quantitative mass spectrometry (MS)-based proteomics analysis of kidney glomeruli or cortex from the mouse models. Our results highlight a conserved (throughout T1D and T2D models and humans) downregulation of peroxisomal function and their cross-talk with mitochondria in early and late DKD, opening up new avenues for DKD therapy.

## 2. Results

### 2.1. Physiopathologic Characterization of Ins2Akita and db/db Mouse Models

Our study was performed using the Ins2Akita mouse model representing early to late kidney morphological lesions and dysfunction as observed in T1D DKD [34]. Further, the db/db mice investigated displayed insulin resistance, obesity, T2D and progressive kidney histological lesions as observed in human T2D [34]. Ins2Akita mice became hyperglycemic at two months of age (Figure S1A) and exhibited a significantly increased urinary albumin to creatinine ratio (ACR) at four months of age (Figure S1A) compared to WT mice. Six-month-old db/db mice displayed hyperglycemia (Figure S1B) and increased ACR compared to the control mice (db/dm) (Figure S1B).

### 2.2. Glomerular Proteome Profiles from Ins2Akita Mice Revealed Prominent Changes in Mitochondrial and Peroxisomal Proteins in Early and Late DKD

Proteomics analysis was applied to investigate the differences in the molecular profiles of glomeruli between Ins2Akita mice at two months of age (INS2) (early DKD) and four months of age (INS4) (late DKD) and respective controls (Table 1). An average of 1550 proteins in wild-type mice and 1600 proteins in Ins2Akita mice were detected (Table S3). The full list of identified proteins using a 55% frequency threshold is shown in Table S4; Sheet 1 (Ins2Akita glomerular dataset).

These protein lists largely overlapped with similar published proteomics datasets (Waanders et al. 2009 [37]). In brief, most of the identified proteins in our study were also detected in these published data (60% for wild-type mice and 73% for the Ins2Akita) [37]. Correlation analysis of the protein abundance detected in our study with Waanders et al. 2009 [37] resulted in correlation factor R = 0.7 for both the wild-type and the Ins2Akita mice (Figure S2), demonstrating consistency between the different studies.

Multiple pair-wise comparisons were performed to identify the consistent changes associated with early DKD (Table S4; Sheet 1).

When comparing the abundance of proteins in two-month-old Ins2Akita to the respective two-month-old WT controls, 100 proteins were significantly upregulated (Table S4; Sheet 2) and 77 were downregulated (Table S4; Sheet 3) in Ins2Akita. Similarly, a comparison of the abundance of proteins between Ins2Akita and WT four-month-old mice revealed 155 proteins with significantly increased abundance (Table S4; Sheet 2) and 111 with decreased abundance in Ins2Akita (Table S4; Sheet 3).

The biological functions represented by the deregulated glomerular proteins associated with early DKD are predominately related to cholesterol metabolic process, glycine metabolic process, lipid storage, cellular amino acid catabolic process, oxidoreductase activity and fatty acid beta-oxidation (Table S5; Sheet 1. Respective pathways are summarized in Table 1). The most prominent biological functions of the proteins significantly changed in late DKD are the branched-chain amino acid catabolic process, glutamate and glutamine metabolic process, lipid storage, carboxylic acid catabolic process, fatty acid beta-oxidation, acyl-CoA metabolic process and oxidoreductase activity (Table S5; Sheet 2. A summary of the main pathways is provided in Table 2).

To verify the observations and identify early changes associated with the disease, glomerular proteins that prominently changed in diabetic animals in early and late stages were studied further. Twenty-five upregulated and 18 downregulated proteins with consistently changed trend between the stages of DKD were selected (Table S4; Sheet 4).

**Figure 1:**
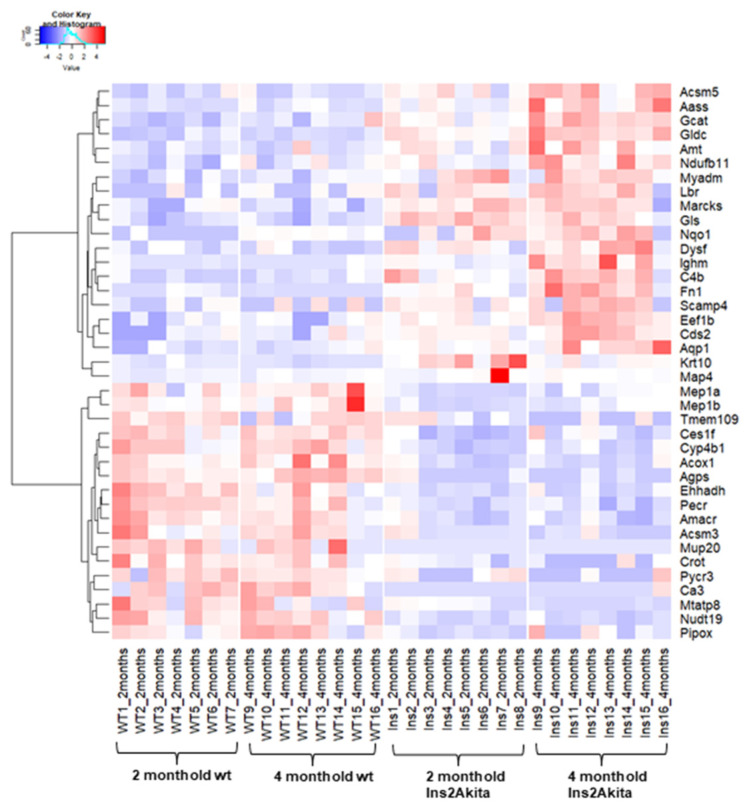
Heatmap of the consistently expressed glomerular proteins in early and late DKD. The expression changes of the 39 (21 up- and 18 downregulated) in early and late DKD versus controls are illustrated in the heatmap for each group, indicating the similar trend of expression of these proteins in the two disease stages.

To correct for any potential impact of aging, the differences from the comparison WT4 vs. WT2 (Table S6) were subtracted from the above glomerular proteins associated with DKD; this gave rise to a final list of 39 proteins (21 up- and 18 downregulated) with consistent expression trends (increased/decreased abundance) in early and late DKD versus controls (Figure 1; Table 3 and Table 4, respectively).

According to the literature, and the databases Uniprot and Human Protein Atlas, among the 21 upregulated proteins, 7 are already related to diabetes (Table 3 and in detail in Table S7) and 15 are attributed to kidney expression and/or function (Table 3 and in detail in Table S7). Among the 18 downregulated proteins, 7 are related to diabetes (Table 4 and in detail in Table S7) and 14 have documented kidney expression and/or function (Table 4 and in detail in Table S7).

The biological functions of the consistently changed glomerular proteins associated with early and late DKD are listed in Table 5.

As shown in Table 5, many of the upregulated proteins are involved in amino acid metabolism performed in mitochondria whereas the downregulated proteins are mainly involved in fatty acid catabolism and ether lipid biosynthesis which are recognized as basic peroxisomal functions.

### 2.3. Validation of Findings in Late T2D DKD Animal Model and Cross-Omics Validation, Further Confirming Peroxisomal Changes in DKD

In order to validate the findings of the glomerular proteome analysis and investigate further if they are also observed in a T2D DKD model, the kidney cortex proteomic profile of db/db mice, a model of T2D DKD, of advanced age (6 months old) was also analyzed. The diabetic stage (late DKD) of the db/db mouse model was verified via biochemical data analysis (Figure S1). Multiple pair-wise comparisons were performed (described below).

On average 674 proteins were detected in wild-type mice and 670 in db/db mice (Table S8). A comprehensive list of the identified proteins including the fold change (ratio) and the statistics is shown Table S9; Sheet 1 (db/db kidney cortex dataset).

When comparing the db/db to respective WT data, 33 upregulated proteins (Table S9; Sheet 2), the majority related to glutamine and glutamate metabolic process, and 23 downregulated proteins (Table S9; Sheet 3) mostly related to peroxisomal protein import, were identified. Interestingly, these processes were also observed in early T1D DKD (Section 2). Taken together, these observations suggest that common deregulated processes between early T1D, late T1D and T2D DKD exist.

Several of the proteins associated with DKD identified in glomeruli from T1D mice were also found in the kidney cortex from T2D mice (Table 6). Specifically, NUDT19, AMACR and PIPOX were downregulated in DKD in both glomeruli and the kidney cortex in T1D and T2D models, respectively (Table 6).

To further place our findings of deregulated mitochondrial and peroxisomal proteins in the context of published datasets in DKD, kidney transcriptomics data from DKD patients vs. healthy controls and DKD mouse versus healthy controls were retrieved from the Nephroseq database (7 datasets in total; [38,39,40]). In addition, a reported single-cell kidney transcriptomic dataset of early human DKD [41] was also investigated.

**Table 6:** Differentially expressed proteins with consistent expression trend in glomeruli and cortex in early and late DKD. Differentially expressed proteins with consistent expression trend in glomeruli and cortex collected from Ins2Akita and db/db mice, respectively, at different ages (months 2, 4, 6). The downregulated proteins are presented in red color.

Description	Symbol	Ratio			Transcriptomics Expression (Nephroseq; in DKD vs. Controls) (Ref.)	Single-Cell Human Kidney Transcriptomics Expression (Wilson et al. [41])
		db/db vs. WT Kidney Cortex 6 Months	Ins2Akita vs. WT Glomeruli 2 Months	Ins2Akita vs. WT Glomeruli 4 Months		
Nucleoside diphosphate-linked moiety X	NUDT19	0.236	0.25	0.23	Decrease [40]	not detected
Peroxisomal sarcosine oxidase	PIPOX	0.406	0.567	0.615	Decrease [39]	not detected
Alpha-methylacyl-CoA racemase	AMACR	0.185	0.52	0.56	Decrease [39]	decrease

Of the reported differentially expressed and ‘consistent’ (Table 6) mitochondrial and peroxisomal proteins, mRNA expression levels were recorded for the three peroxisomal proteins NUDT19, PIPOX and AMACR with a reported decrease in DKD cases versus controls (Table 6).

The cross-omics analysis highlighted additional glomerular-cortical mitochondrial and peroxisomal proteins (Table 3, Table 4, Tables S10 and S11) in agreement in their change in mRNA and protein levels. In the transcriptomics datasets, mRNA expression levels were also recorded for three mitochondrial proteins (GLS, GLDC, AMT) and five peroxisomal proteins (ACOX1, CROT, EHHADH, AGPS and PECR), all consistently changing in early and late DKD (Table 3 and Table 4), and three peroxisomal (CAT, EPHX2, DAO) cortical proteins associated with late DKD. For all these, with the exception of AMT, changes at the mRNA levels in DKD cases vs. healthy controls were in agreement with the observed changes in protein levels in our animal model experiments (Tables S10 and S11).

### 2.4. IHC Validation of Peroxisomal Deregulation in Human DKD Patients

Given the consistency of these findings, a set of tissue sections of human kidney tissues was analyzed for the expression of the peroxisomal proteins NUDT19, AMACR and AGPS. In addition, the CAT protein that was found downregulated in the cortex of the late DKD animal models (db/db mice) (Tables S10 and S11) was investigated as a control since it is the most studied and best-characterized peroxisomal antioxidant enzyme in kidney disease [42].

**Figure 2:**
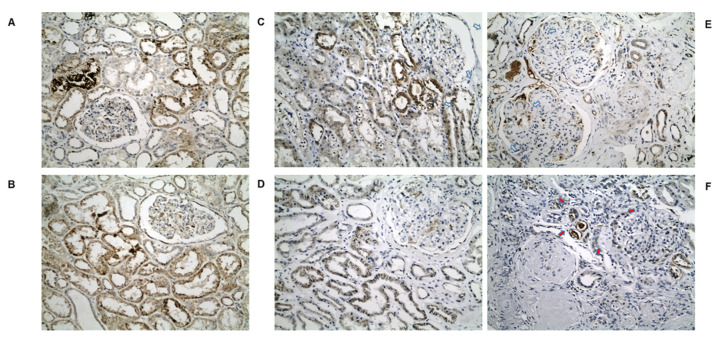
(**A**,**B**): Representative photos of NUDT19 staining in normal kidney tissue (×200). Coarsely or fine granular and/or pale homogeneous cytoplasmic pattern of staining in all types of tubules and some glomerular cells. Representative photos of NUDT19 staining in (**C**) class I, (**D**) class IIb, (**E**) class III and (**F**) class IV DKD (×200). Gradual decrease from class I to IV can be seen. Note the intensification of podocytic staining around Kimmelstiel–Wilson nodules (red arrows).

In a detailed qualitative analysis, NUDT19 in normal kidney tissue (Figure 2A,B) showed tubular staining with a cytoplasmic either coarsely granular or fine granular or pale homogeneous pattern. NUDT19 was detected in all types of tubules. In glomeruli, staining was found mainly in the parietal cells of the Bowman’s capsule and occasionally in podocytes and mesangial cells (Figure 2A). In diabetic patients, NUDT19 staining (Figure 2C–F) was seen in the kidney tubules of all cases, however, reduced compared to controls and with a decrease, in terms of both intensity and extension, from histological class I to class IV. Moreover, staining seemed more prominent in non-proximal convoluted tubules. Although NUDT19 was found only occasionally in the podocytes of the controls, podocytic expression (especially surrounding Kimmelstiel–Wilson nodules) was more intense in advanced cases of DKD.

**Figure 3:**
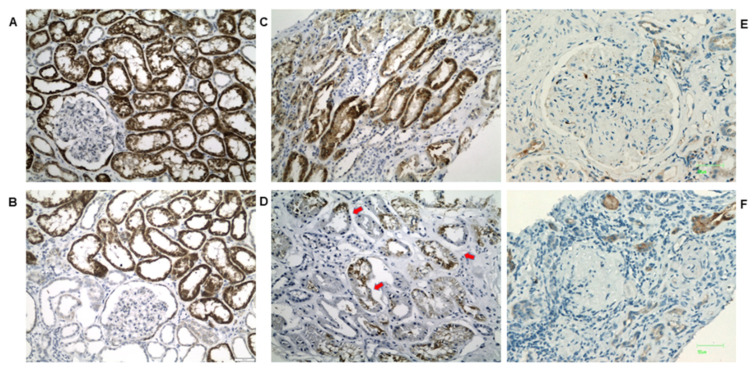
(**A**,**B**): Representative photos of AMACR staining in normal kidney tissue (×200). Cytoplasmic staining, diffuse and strong in PCTs vs. focal, and weak in DCTs. Positivity in some parietal cells is also observed. Representative photos of AMACR staining in (**C**) class I, (**D**) class IIb, (**E**) class III and (**F**) class IV DKD (×200). Gradual loss of expression, usually in all the cells of any given tubule is observed. However, areas of segmental loss of staining can also be noted (red arrows).

AMACR in normal kidney tissue (Figure 3A,B) showed a diffuse, strong and densely granular cytoplasmic staining in almost all proximal convoluted tubules (PCT), while distal convoluted tubules (DCT) demonstrated a focal weak granular cytoplasmic staining (Figure 3). Glomeruli were mostly negative; however, some staining in parietal cells was also observed. Compared to the controls, DKD cases showed a significant decrease in AMACR staining intensity. AMACR staining also gradually decreased from histological class I to class IV DKD (Figure 3C–F). The loss of AMACR expression was more often recorded as a loss of staining in all the epithelial cells of any given tubule. However, in some tubules a segmental loss of expression was seen, with some epithelial cells retaining their cytoplasmic staining.

**Figure 4:**
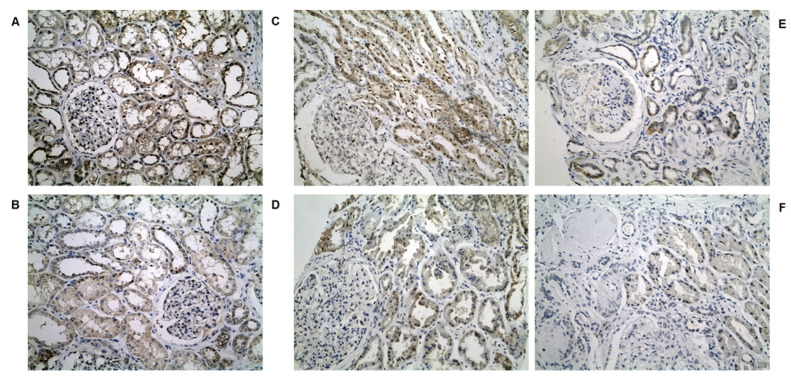
(**A**,**B**): Representative photos of AGPS staining in normal kidney tissue (×200). Diffuse staining of moderate intensity with a cytoplasmic, membranous and occasionally nuclear pattern in most tubules and a few parietal cells. Representative photos of AGPS staining in (**C**) class I, (**D**) class IIb, (**E**) class III and (**F**) class IV DKD (×200). Decrease in expression compared to controls. Occasional nuclear pattern can still be noted in some cases.

In normal kidney tissue, AGPS showed a diffuse staining of moderate intensity in most kidney tubules with a cytoplasmic, membranous and occasionally nuclear pattern (Figure 4A,B). In glomeruli, focal staining in parietal cells was seen. DKD cases exhibited a definite loss of AGPS expression irrespectively of the DKD histological class (Figure 4C–F). Of note, nuclear staining remained in some tubular epithelial cell nuclei.

**Figure 5:**
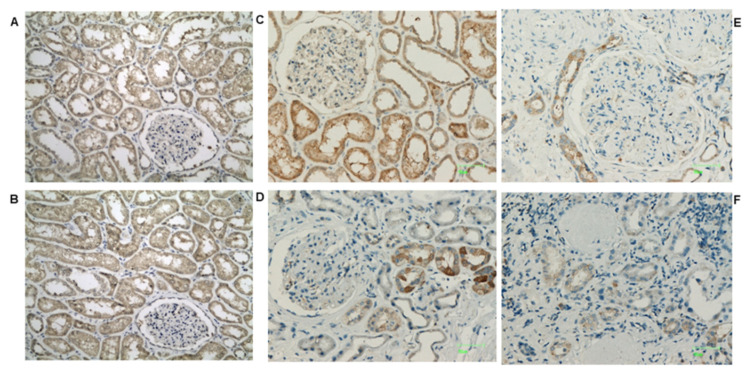
(**A**,**B**): Representative photos of CAT staining in normal kidney tissue (×200). Diffuse, cytoplasmic staining of moderate intensity in tubules and parietal cells. Representative photos of CAT staining in (**C**) class I, (**D**) class IIb, (**E**) class III and (**F**) class IV DKD. Loss of CAT expression compared to controls. CAT positivity in endothelial cells of the capillary walls is observed in some cases (**D**,**E**).

Controls showed diffuse CAT staining in all types of renal tubules with a cytoplasmic, partly finely granular pattern of moderate intensity (Figure 5A,B). In glomeruli, CAT staining was restricted to parietal epithelial cells. No endothelial cell staining was seen in vessels or glomerular capillaries. DKD cases demonstrated a definite loss of CAT expression in comparison with the controls (Figure 5C–F). Interestingly, in glomeruli of DKD cases, CAT was detected in the endothelial cells of the capillary walls, suggesting a possible endothelial activation under pathological conditions. However, no endothelial cell expression in extraglomerular vessels was seen. Another observation was a preferential CAT expression in tubules surrounding globally sclerosed glomeruli.

**Figure 6:**
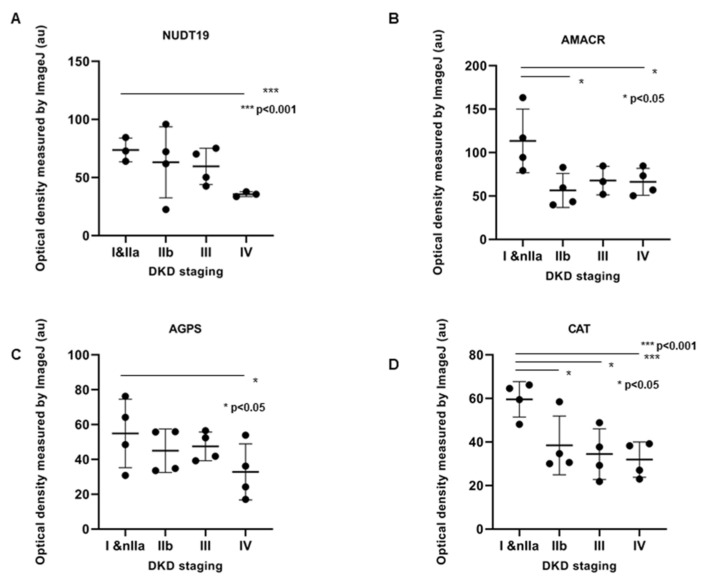
Quantitative analysis of the IHC staining intensity using ImageJ software. Relative quantification scores revealing a decrease in NUDT19 (**A**), AMACR (**B**), AGPS (**C**) and CAT (**D**) among different DKD stages. Protein expression among groups I+IIa compared to group IV for each of the aforementioned proteins. Values are means ± SD. (*n* = 4 cases of stage I and IIa, 4 cases of stage IIb, 4 cases of stage III and 4 cases of stage IV)(Student’s *t*-test), *** *p* < 0.001; * *p* < 0.05.

Statistical significance among different groups was confirmed with ANOVA analysis (*p* < 0.05) for CAT (*p* = 0.0002), AMACR (*p* = 0.0005) and AGPS (*p* = 0.05), whereas in the case of NUDT19, a *p* > 0.05 was received. The latter may be attributed to the lower sample size among different groups for NUDT19 compared to the other proteins. However, it appears that the intensity of the protein stain in IHC, as shown in Figure 2, Figure 3, Figure 4 and Figure 5, follows the trend of the proteomics results obtained from the DKD animal model samples. The comparison of each group of samples for CAT, AMACR, AGPS and NUDT19 applying a Student’s *t*-test showed a statistically significant difference among groups I+IIa compared to group IV for each of the aforementioned proteins (Figure 6). A control group (no DKD) was not included in the statistical analysis due to its small sample size (*n* = 1).

## 3. Discussion

This study aimed to investigate proteomic changes associated with early DKD and its progression. Proteomic data were generated from two well-characterized mouse models (Ins2Akita) of diabetes T1D and (db/db) of T2D. Our study was mainly focused on glomerular proteins that are consistently changed in T1D animals in early and late DKD. The expression of these glomerular proteins was further investigated in the kidney cortex proteome of db/db mice. Multiple consistent changes were observed in proteins involved in cholesterol biosynthesis, mitochondrial respiratory chain function, peroxisomal function and amino acid metabolism.

The observed elevated levels of mitochondrial enzymes are in agreement with the existing literature: GLS (glutaminase kidney isoform) catalyzes the first reaction in the kidney catabolism of glutamine [43]. Associations of incident prediabetes or T2D with higher levels of glutamate were reported previously [44,45,46,47,48]. GLDC (glycine dehydrogenase-decarboxylating) and AMT (aminomethyltransferase) participate in mitochondrial glycine cleavage in the kidney [49]. Low levels of glycine are related to diabetes and could potentially predict future T2D [48,50,51,52]; they may also reflect glycine utilization towards glutathione production to counteract oxidative stress [53]; and/or an increased uptake of glycine by insulin-resistant tissues to support gluconeogenesis [54]. GCAT (2-amino-3-ketobutyrate coenzyme A ligase) participates in the degradation of L-threonine to glycine. Interestingly, decreased threonine levels are reported in diabetes [55]. AASS (alpha-aminoadipic semialdehyde synthase) catalyzes the first two steps in lysine degradation. Of note, lysine levels in the plasma and serum of T2D patients are lower in comparison to controls [45]. Mitochondrial enzymes involved in branched-chain amino acid catabolism are elevated in glomeruli of late DKD in agreement with previous studies [56].

The observed changes in peroxisomal proteins, the most prominent observed alteration in our study, are in general agreement with earlier reports [22,57], which suggested the decreased expression of key peroxisomal enzymes and regulators of fatty acid oxidation (FAO) in CKD or DKD compared to healthy kidneys.

Peroxisomal enzymes shorten the long chain of very long-chain fatty acids (VLCFA), which are then oxidized to acetyl-CoA by -acyl-CoA oxidase (ACOX) [58], and subsequently converted to acyl-carnitine by the carnitine octanoyltransferase (CROT) [59]. Interestingly, the measurements of CAT, ACOX and CROT in the kidney of db/db mice revealed significantly reduced levels (to approximately 2/3) of these peroxisomal enzymes in comparison to controls [60] in line with our results, which is potentially related to the well-established accumulation of lipids in the kidneys of diabetic humans and experimental animals [61,62].

EHHADH (enoyl-CoA hydratase and 3-hydroxyacyl CoA dehydrogenase) [63] was recently shown to oxidize medium- and long-chain fatty acids [64,65]. In line with our findings, decreased mRNA levels of EHHADH were detected in human DKD glomeruli tissues [66].

Our study also indicated decreased levels of AGPS in mouse proteomics and in human IHC analyses of DKD tissues, with AGPS levels decreasing with DKD progression. AGPS is the main peroxisomal enzyme mediating (bio)synthesis of plasmalogens [67], which act as antioxidants [68] and bile acids. Low levels of plasmalogens have been earlier observed in both T1D [69,70] and T2D [71].

Decreased levels of PIPOX (peroxisomal sarcosine oxidase) were observed in our diabetic mice. PIPOX lowers pipecolate accumulation through oxidation and increases synthesis of glutaryl-CoA [72,73]. In line with our findings, previous studies detected increased levels of pipecolate in T1D mice compared to healthy controls [61]. Further, decreased mRNA levels of PIPOX were also reported in human DKD glomerular tissues [66].

Our study indicated decreased levels of AMACR, which is involved in the bile acid biosynthesis pathway ([74] in DKD mouse and human kidney tissues. This finding may be linked to the earlier observed impairment of bile acid synthesis in T2D [75].

Decreased levels of NUDT19 were also observed in our diabetic mice as well as in human DKD tissues. NUDT19 degrades and regulates CoA in the kidneys [76], thus, regulating the peroxisomal CoA pool and b-oxidation [77].

Interestingly, in the cortex of db/db mice of late DKD, three additional downregulated peroxisomal proteins were detected: CAT, EPHX2 (bifunctional epoxide hydrolase 2) and DAO (D-amino acid oxidase; also known as DAAO). CAT is an antioxidant enzyme [42] whose expression levels also decreased with DKD progression in our human IHC analyses. CAT has also been found downregulated in kidneys and serum from STZ (T2D induced) rodents [78]. Interestingly, aberrant catalase activity has been found to increase mitochondrial oxidative stress in kidney proximal tubules [79]. EPHX2 (an antioxidant enzyme) protein and mRNA levels, in accordance with our study, were found decreased in the kidneys of streptozotocin (STZ)-induced diabetic mice [80] as well as rodents of progressive kidney disease [81]. Finally, DAO (participating in amino acid degradation [82]) expression was also decreased in the kidney of DKD alloxan-diabetic rats, in line with our results [83].

## 4. Conclusions

**Figure 7:**
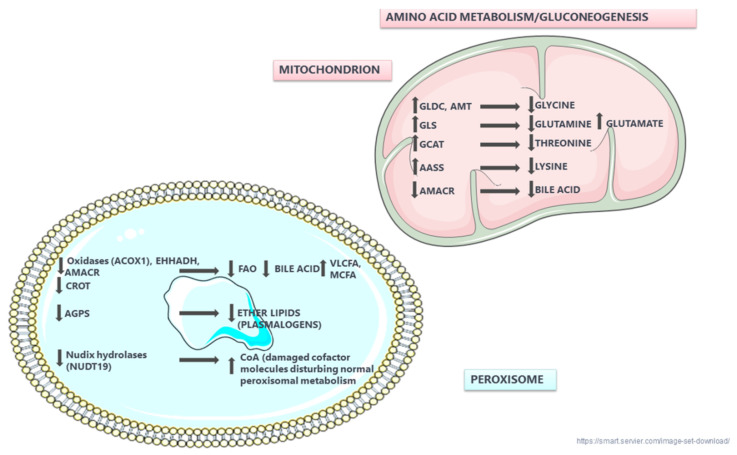
Peroxisomal and mitochondrial dysfunction in DKD based on consistently changed proteins in our study. It may be hypothesized that the observed decreased expression of peroxisomal proteins involved in lipid metabolism and CROT leads to elevated levels of VLCFA and MCFA, reduced bile acid synthesis and disruption of acylcarnitines transportation to mitochondria. Moreover, downregulation of AGPS reduces plasmalogens synthesis. Peroxisomal Nudix hydrolases, such as NUDT19 (hydrolyzes CoA), clean the cell from harmful metabolites such as ROS, indicating their role as ‘housecleaning’ enzymes. In mitochondria, increased expression of GLDC, GLS, GCAT and AASS favors gluconeogenesis.

Collectively, our study highlights and brings together multiple protein changes, which had to a good extent been observed at the mRNA level in animal models or human tissue, supporting, as a step further, their role at an early time point in DKD development. Based on our results, a massive disruption of the mitochondrial–peroxisome cross-talk in early DKD is supported (summarized in Figure 7) with special emphasis on fatty acid oxidation. This is of great interest since most of the peroxisome studies in DKD are focused mainly on the mitochondrial oxidative stress and the role of peroxisomal catalase [42]. As a next step, we plan to validate our results in a large cohort of human DKD kidney samples in order to unveil the molecular details of mitochondrial–peroxisomal cross-talk in DKD, which remain unclear. Moreover, functional assays or metabolism analysis such as peroxisomal and mitochondrial β-oxidation, measures of lipid uptake and ATP levels are included in our future plans.

## 5. Materials and Methods

### 5.1. Animals

The mouse models C57BL/6-Ins2Akita/J (Ins2Akita-T1D) and BKS.Cg-+Leprdb/+Leprdb/OlaHsd (dbdb-T2D) were used. In the glomerular proteome study 4 groups of animals were included, 2-month-old Ins2Akita (INS2) (*n* = 8) and respective controls (WT2; *n* = 7); and 4-month-old Ins2Akita (INS4) (*n* = 8) and respective controls (WT4, *n* = 8) [15]. For the kidney cortex proteome study, db/db mice were used. The former included 6-month-old db/db (*n* = 3) and respective controls db/dm: (*n* = 5).

### 5.2. Isolation of Glomeruli

Isolation of glomeruli was conducted as previously described [15]. In brief, the aorta of anesthetized mice was catheterized and perfused with 40 mL of a Dynabeads M-450 Tosylactivated suspension (4.5 µm diameter, Dynal A.S., Oslo, Norway) at 2 × 10^6^ beads/mL followed by a perfusion of cold PBS (15 mL). The kidneys were pressed through a cell strainer (70 µm) and washed with cold PBS (20 mL). After centrifugation (200× *g* for 5 min at 4 °C), the pellet was resuspended in PBS (2 mL) in an Eppendorf tube and Dynabead-loaded glomeruli were pelleted using a concentrator (Dynal A.S., Oslo, Norway). The pellet was washed with PBS (5 × 1 mL) and resuspended in 100 µL PBS, resulting in the enriched glomerular suspension (~ 4000 per kidney). This protocol allows to obtain a relatively high purity level of isolated glomeruli based on microscopy observation and enrichment in glomerular-specific genes [15].

### 5.3. Murine Kidney Histology

Kidney histology was performed as described in Ins2Akita mice [15] by Klein et al., 2020, and in db/db mice (Figure S1). In brief, kidney lesions were assessed by specific (immuno) histological evaluation of the kidney structure (PAS) and fibrosis (glomerulo and tubulointerstitial fibrosis, Masson-trichrome and Sirius red staining, collagen III staining). Quantifications resulted from the analysis of at least 50 glomeruli.

### 5.4. Biochemical Analysis

Albumin concentration in urine was determined using the AlbuWell kit (WAK-Chemie Medical GmbH, Steinbach, Germany). Creatine concentration was determined using the Jaffe method. Glucose concentration in blood was determined using a glucometer.

### 5.5. Sample Preparation for Proteomics

Sample preparation was performed as previously described [84]. Briefly, samples were homogenized in lysis buffer (7 M Urea, 2 M Thiourea, 4% CHAPS and 1% DTE) and processed with the GeLC–MS method [85]. Ten micrograms of each sample were loaded in SDS-PAGE (5% stacking, 12% separating) and the electrophoresis was stopped when the samples entered the separating gel. A fixation step (30% methanol, 10% acetic acid) for 30 min was performed and the gels were washed with water (3 × 5 min washes) prior to colloidal Coomassie Blue staining (overnight). Another series of water washes (3 × 5 min washes) was performed to remove the excess stain. All bands were excised from the gel and sliced into small pieces (1–2 mm). Gel pieces were destained with destain solution (40% acetonitrile, 50 mM NH_4_HCO_3_) followed by reduction (10 mM DTE in 100 mM NH_4_HCO_3_) for 20 min RT and alkylation (54 mM iodoacetamide in 100 mM NH_4_HCO_3_) for 20 min RT in the dark. A series of washes was performed for 20 min at RT with the following buffers: 100 mM NH_4_HCO_3_, destaining solution (40% acetonitrile, 50 mM NH_4_HCO_3_), ultra-pure water. Gel pieces were dried in a Speed Vac and trypsinized overnight in the dark at RT. For the trypsinization process, 600 ng trypsin per sample was utilized (trypsin stock solution: 10 ng/μL in 10 mM NH_4_HCO_3_, pH 8.5). After trypsinization, the peptide extraction was performed with subsequent incubations of the gel pieces with the following buffers: 50 mM NH_4_HCO_3_ for 15 min RT, 5% formic acid, 50% acetonitrile for 15 min RT (the latter was repeated once). Extracted peptides were eluted in a final volume of 600 μL and cleaned with 0.22 μm PVDF filters (Merck, Darmstadt, Germany). After cleaning, the tryptic peptides were placed in a Speed Vac to dry. Dried peptides were resuspended in mobile phase A (0.1% formic acid, pH 3.5) and subjected to LC–MS/MS analysis.

### 5.6. LC–MS/MS Analysis

A Dionex Ultimate 3000 UHPLC system coupled with the high-resolution nano-ESI Orbitrap-Elite mass spectrometer (Thermo Fisher Scientific, Waltham, MA, USA) was utilized for the LC–MS/MS analysis. Each sample was resuspended in 10 μL mobile phase A. Injection volume for the LC–MS/MS analysis was 5 μL. Samples were loaded on an Acclaim PepMap 100, 100 μm × 2 cm C18, 5 μm, 100 Ȧ trapping column with the ulPickUp Injection mode at a flow rate of 5 μL/min. Peptides were separated in an Acclaim PepMap RSLC, 75 μm × 50 cm, nanoViper, C18, 2 μm, 100 Ȧ column retrofitted to a PicoTip emitter. Mobile phase A (aqueous: 0.1% formic acid, pH 3.5) and B (organic: 100% acetonitrile, 0.1% formic acid) were used for a multi-step gradient elution. The peptides were eluted with a 240 min LC gradient starting from 2% B and rising to 80% B with a flow rate of 300 nL/min and a column temperature at 35 °C. Gaseous phase transition of the separated peptides was achieved with positive ion electrospray ionization applying a voltage of 2.5 kV. In every MS survey scan, the top 10 most abundant multiply charged precursor ions (m/z 300–2200) with an intensity threshold of 500 counts were selected and fragmented with the HCD method. Mass resolution was 60,000 in MS and 15,000 in MS/MS. Normalized collision energy was set to 33 and already targeted precursors were dynamically excluded for further isolation and activation for 45 sec with 5 ppm mass tolerance.

### 5.7. MS Data Processing

Raw files were analyzed with the Proteome Discoverer 1.4 software package (Thermo Fisher Scientific, Waltham, USA), using the SEQUEST search engine and the UniProt mouse (*Mus musculus*) reviewed database, downloaded on 22 November 2019, including 16.935 entries. Stringent criteria were used for protein identification and relative quantification as previously established in our lab [86]. Cysteine carbamidomethylation was used as the fixed modifications and methionine oxidation as the variable modifications. Two missed cleavage sites were allowed, and the precursor and fragment mass tolerance were set at 10 ppm and 0.05 Da, respectively. The False Discovery Rate was 1% (peptide level). Label free quantification analysis was performed considering the precursor ion area values that were exported from the total ion chromatogram as defined by the Proteome Discoverer 1.4 software package.

Output files from the Proteome Discoverer were processed with an in-house script in the R environment for statistical computing (version 4.0.3) as follows: Protein lists were concatenated into a master table. Raw protein intensities for each individual sample were subjected to normalization according to X’ = X/Sum(Xi) ∗ 10^6^ and only proteins present in at least 55% of the samples in at least one group were further selected for downstream statistical analysis.

### 5.8. Functional Analysis

Pathway annotation was performed with the ClueGO plug-in 3.7.2 (Cytoscape) using the REACTOME pathway database (updated on 17 February 2020). The same GlueGO plug-in was used for the biological function annotation with the addition of the GO-Biological Process EBI-Uniprot GOA database (updated on 17 February 2020). Statistically significant pathways corrected for multiple testing (Benjamini–Hochberg (BH) corrected *p*-value ≤ 0.05, two-sided hypergeometric test) were further considered. Results were interpreted based on biological relevance by utilizing only the leading term from each group.

### 5.9. Investigation through Transcriptomics Data Analysis

Nephroseq (www.nephroseq.org, accessed on 30 November 2021) was employed for the investigation of the expression of the shortlisted mitochondrial and peroxisomal proteins in existing mouse and human transcriptomics datasets. The list of proteins was uploaded in Nephroseq v4 in the form of EntrezGene IDs. DKD datasets selection was held after application of the filters: Primary Filters > Group > Diabetic nephropathy. The corresponding gene expression was searched in seven available DKD mouse and human datasets observed after filtering, on comparison of DKD vs. Healthy Living Donor groups (Table S1). Only significantly deregulated genes (*p* < 0.05) were extracted, and their differential expression was compared with the mitochondrial and peroxisomal deregulated proteins.

### 5.10. Clinical Material

Within a period of eight years (2013–2020) a total number of 100 kidney biopsies diagnosed as DKD were retrieved from the human Renal Biopsies archive of the 1st Department of Pathology of Athens (National and Kapodistrian University of Athens, Medical School, Greece). All cases were initially classified based on their glomerular lesions into the four classes of DKD (I, IIa/b, III and IV) according to Tervaert et al. [87]. Interstitial fibrosis and tubular atrophy (IFTA), as well as interstitial inflammatory infiltration and vascular lesions (arteriolar hyalinosis, arteriosclerosis), were also studied and scored from 0 to 9 according to Tervaert et al. [87] in order to assess the severity and chronicity of DKD.

Information on gender, age, serum creatinine value and albuminuria levels was collected, and the estimated Glomerular Filtration Rate (eGFR) was calculated using the online National Kidney Foundation GFR Calculator [88]. Combining eGFR and albuminuria levels, the cases were classified to 5 clinical stages (G1, G2, G3a/b, G4 and G5–A1, A2 and A3) of chronic kidney disease (CKD) due to diabetes mellitus (DM) according to KDIGO Guidelines [89]. All cases were classified as A3 since albuminuria levels were always >0.3 g/24 h.

Out of the 100 cases, 16 were selected in order to assess the immunohistochemical expression of the peroxisomal proteins Nucleoside diphosphate-linked moiety X motif 19 (NUDT19), alpha-methylacyl-CoA racemase (AMACR), peroxisomal catalase (CAT) and alkyldihydroxyacetonephosphate synthase (AGPS) in human renal tissues with DKD.

The selected cases met the following criteria: (1) Absence of coexisting non diabetic kidney disease, (2) Adequate representation of the histopathological lesions of DKD, reflecting the 4 histological classes of DKD and (3) kidney function deterioration, as it is reflected in the CKD stage, equivalent to DKD lesion progression.

Additionally, normal kidney tissue showing no signs of DKD, CKD or other kidney pathology obtained from radical nephrectomy specimens were used as controls.

The main clinicopathological parameters of the 16 cases are shown in Table S2.

### 5.11. Immunohistochemistry of Human DKD Specimens

Immunohistochemical detection of the examined proteins was performed on 4-μm-thick formalin-fixed paraffin sections which underwent overnight heating at 37 °C, deparaffinization, rehydration and antigen retrieval using an automated module (PT Link, Dako) for 20 min at 96 °C with the reagents EnVision FLEX Target Retrieval Solution High pH (50×) (Dako, DAKO EnVision kit, DAKO, Carpinteria, CA) for CAT and EnVision FLEX Target Retrieval Solution Low pH (50×) (Dako) for NUDT19, AGPS and AMACR. To block endogenous peroxidase activity, 0.3% hydrogen peroxide in Tris-buffered saline (TBS) was applied for 15 min. Sections were rinsed with TBS and normal horse serum was applied for 20 min to prevent non-specific antibody binding. This step was followed by overnight incubation of the sections at 4 °C with the primary antibodies: anti-AMACR (rabbit polyclonal) (Atlas Antibodies, Stockholm, Sweden) at a dilution 1:2000, anti-AGPS (rabbit polyclonal) (Atlas Antibodies, Sweden) at a dilution 1:50, anti-CAT (rabbit polyclonal) (Atlas Antibodies, Sweden) at a dilution 1:3000 and anti-NUDT19 (EPR13162-63) (rabbit monoclonal) (abcam, Cambridge, UK) at a dilution 1:100. For visualization, a two-step technique (polymer, HRP-conjugated; Vector Laboratories, Burlingame, CA, USA) was used with diaminobenzidine as a chromogen. Haematoxylin was used to counterstain the sections.

### 5.12. Evaluation of Immunohistochemistry

Qualitative immunohistochemical evaluation was assessed by two independent observers (HG and DP) blinded to clinical data. The pattern, topography, extension and intensity of staining of all the antibodies under study in both the controls and DKD cases were analyzed. A rough comparison of the expression of each marker between the controls and DKD cases, as well as between cases of different DKD classes was also performed.

Staining intensity was also further quantified using ImageJ software as previously described [85]. Optical density was normalized over the unstained tissue and mean intensity values were estimated. Statistical significance among multiple groups (stages) was confirmed with ANOVA analysis and further pair-wise comparisons were performed with the Student’s *t*-test. Values of *p* < 0.05 were considered as statistically significant.

### 5.13. Statistical Analysis of Proteomics Data

Statistical significance of continuous variables was defined at *p* < 0.05 with the non-parametric Mann–Whitney test. Proteins with *p* value ≤ 0.05 and ratio ≥ 1.5 (upregulated) or ≤0.67 (downregulated) were considered statistically significant and differentially expressed. Dotplots for the mouse physiopathologic characterization were created with functionality from the ggplot2 and ggpubr R packages. Depicted statistical comparisons correspond to independent Μann–Whitney tests. For the correlation analysis, Spearman’s correlation was utilized to assess the relationships of our data to one external proteomics dataset [37], using the means of the normalized protein intensity across the samples after transforming them to the natural logarithmic scale. A heatmap was created with the gplot package, after Z-scaling of the normalized protein intensities. Euclidean distance and Ward’s hierarchical method (option: ward.D2) were selected for the clustering of both rows and columns. Graphing and statistical analysis were performed in the RStudio environment (R version 4.0.3).

## Figures and Tables

**Table 1:** Biological pathways represented by glomerular proteins associated with early DKD. Significant pathways represented by glomerular proteins associated with early DKD according to the BH corrected *p* value of the GO Term. The upregulated (green color) and downregulated proteins (red color) in Ins2Akita compared to WT controls are shown below.

GO Term	Term *p* Value Corrected with BH	Group Genes
Cholesterol biosynthesis	0.008	Acat2, Lbr, Nsdhl
Regulation of ornithine decarboxylase (ODC)	0.011	Nqo1, Psmd11, Psmd3
Metabolism of polyamines	0.014	Nqo1, Psmd11, Psmd3
Peroxisomal protein import	9.05 × 10^−10^	Acox1, Agps, Crot, Dao, Ehhadh, Nudt19, Pecr, Pipox, Scp2
Peroxisomal lipid metabolism	9.54 × 10^−10^	Acox1, Aldh3a2, Crot, Ehhadh, Nudt19, Pecr, Scp2
Protein localization	1.14 × 10^−9^	Acox1, Agps, Aldh3a2, Crot, Dao, Ehhadh, Nudt19, Pecr, Pipox, Scp2
Fatty acid metabolism	1.31 × 10^−6^	Acox1, Aldh3a2, Crot, Cyp4b1, Ehhadh, Ggt1, Nudt19, Pecr, Scp2

**Table 2:** Biological pathways represented by glomerular proteins associated with late DKD. Significant pathways represented by glomerular proteins associated with late DKD according to BH corrected *p* value of the GO Term. The upregulated (green color) and downregulated proteins (red color) in Ins2Akita compared to WT controls are shown below.

GO Term	Term *p* Value Corrected with BH	Group Genes
Metabolism of amino acids and derivatives	1.41 × 10^−7^	Aass, Acad8, Aldh4a1, Amt, Cth, Dmgdh, Gcat, Gcsh, Gldc, Gls, Glud1, Gstz1, Hibadh, Mccc1, Nqo1, Prodh, Sardh
HDL remodeling	0.0005	Alb, Apoa1, Apoe
Glyoxylate metabolism and glycine degradation	0.0008	Aldh4a1, Amt, Gcsh, Gldc
Branched-chain amino acid catabolism	0.002	Acad8, Hibadh, Mccc1
The citric acid (TCA) cycle, respiratory electron and ATP synthesis by chemiosmotic coupling	0.006	Atp5h, Gstz1, Ndufb11, Ndufb6, Ndufb7, Ndufs5
Protein localization	4.83 × 10^−8^	Acot4, Acox1, Agps, Atad1, Crot, Dhrs4, Ehhadh, Ldhd, Nudt19, Pecr, Pipox
Peroxisomal protein import	7.25 × 10^−8^	Acot4, Acox1, Agps, Crot, Dhrs4, Ehhadh, Nudt19, Pecr, Pipox
Peroxisomal lipid metabolism	1.93 × 10^−6^	Clasp2, Clip1, Dync1i2, Pafah1b1, Smc3
Fatty acid metabolism	0.0007	Acot4, Acox1, Crot, Cyp4b1, Ehhadh, Nudt19, Pecr, Ptgs1
Beta-oxidation of very long-chain fatty acids	0.0007	Acot4, Acox1, Ehhadh

**Table 3:** Consistently upregulated glomerular proteins in early and late DKD. Twenty-one consistently upregulated glomerular proteins in early and late DKD versus controls. Of these proteins, 7 are related to diabetes (the references are presented in Table S7) and 15 are attributed to kidney expression and/or function (the references are presented in Table S7).

Accession	Name	pval_INS2vs.WT2	Ratio_INS2vs.WT2	pval_INS4vs.WT4	Ratio_INS4vs.WT4	Related to Diabetes	Kidney Expression and/or Function
D3Z7P3	Glutaminase kidney isoform, mitochondrial OS	0.000311	2.94	0.000311	2.33	YES	YES
Q91W43	Glycine dehydrogenase (decarboxylating), mitochondrial	0.000311	2.16	0.000155	2.88	YES	YES
P02535	Keratin, type I cytoskeletal 10	0.00124	4.12	0.000155	3.62	NO	NO
O88986	2-amino-3-ketobutyrate coenzyme A ligase, mitochondrial	0.00214	3.54	0.00295	2.57	YES	YES
Q99K67	Alpha-aminoadipic semialdehyde synthase,	0.00218	1.76	0.00295	2.57	YES	YES
P26645	Myristoylated alanine-rich C-kinase substrate	0.00314	2.21	0.00986	2.58	NO	YES
P01029	Complement C4-B	0.00897	5.57	0.00295	4.42	NO	NO
Q99L43	Phosphatidate cytidylyltransferase 2	0.0125	2.12	0.00295	1.92	NO	YES
P27546	Microtubule-associated protein 4	0.0128	6.06	0.0148	1.73	NO	YES
Q02013	Aquaporin-1	0.0128	2.23	0.00187	2.24	NO	YES
Q9JKV5	Secretory carrier-associated membrane protein 4	0.0173	2.065	0.033	2.59	NO	YES
P01872	Ig mu chain C region	0.0231	4.94	0.00147	14.0	NO	NO
Q3U9G9	Lamin-B receptor	0.0231	3.59	0.00817	2.284	NO	NO
O09111	NADH dehydrogenase [ubiquinone] 1 beta	0.0289	1.898	0.00699	1.94	YES	
Q8BGA8	Acyl-coenzyme A synthetase ACSM5, mitochondrial	0.0289	1.52	0.000622	1.95	NO	YES
P11276	Fibronectin	0.0292	3.05	0.000554	103	YES	YES
O35682	Myeloid-associated differentiation marker	0.0321	2.85	0.00699	2.25	NO	NO
O70251	Elongation factor 1-beta	0.0321	1.76	0.00135	2.30	NO	YES
Q9ESD7	Dysferlin	0.0321	2.16	0.00377	4.55	NO	YES
Q8CFA2	Aminomethyltransferase, mitochondrial	0.04	2.17	0.00295	2.95	YES	YES
Q64669	NAD(P)H dehydrogenase [quinone] 1	0.043	6.96	0.01	1.60	NO	YES

**Table 4:** Consistently downregulated glomerular proteins in early and late DKD. Eighteen consistently downregulated glomerular proteins in early and late DKD. Of these proteins, 7 are related to diabetes (the references are presented in Table S7) and 14 are attributed to kidney expression and/or function (the references are presented in Table S7).

Accession	Name	pval_INS2vs.WT2	Ratio_INS2vs.WT2	pval_INS4vs.WT4	Ratio_INS4vs.WT4	Related to Diabetes	Kidney Expression and/or Function
Q99MZ7	Peroxisomal trans-2-enoyl-CoA reductase	0.000311	0.485	0.00466	0.565	NO	YES
Q9DBM2	Peroxisomal bifunctional enzyme	0.000311	0.453	0.01	0.664	NO	YES
O09174	Alpha-methylacyl-CoA racemase	0.000622	0.52	0.00187	0.56	YES	YES
P11930	Nucleoside diphosphate-linked moiety X motif	0.000622	0.2499	0.000311	0.23	NO	YES
Q61847	Meprin A subunit beta	0.000622	0.408	0.00187	0.455	YES	YES
Q9DC50	Peroxisomal carnitine O-octanoyltransferase	0.00124	0.413	0.0122	0.296	YES	YES
Q5FW60	Major urinary protein 20	0.00215	only in wt	0.0324	only in wt	NO	NO
Q64462	Cytochrome P450 4B1	0.00218	0.387	0.00109	0.473	NO	NO
Q91WU0	Carboxylesterase 1F	0.00373	0.387	0.00466	0.579	NO	YES
Q9D826	Peroxisomal sarcosine oxidase	0.005905	0.566932	0.0499	0.615	YES	YES
Q9R0H0	Peroxisomal acyl-coenzyme A oxidase 1	0.00591	0.599	0.00109	0.496	YES	YES
P16015	Carbonic anhydrase 3	0.00721	only in wt	0.0071	0.119	NO	NO
Q3UBX0	Transmembrane protein 109	0.014	0.590	0.0289	0.289	NO	YES
Q8C0I1	Alkyldihydroxyacetonephosphate synthase, peroxisomal	0.0157	0.297	0.000682	0.08254	YES	YES
P03930	ATP synthase protein 8	0.0173	0.353	0.0287	0.0285	NO	NO
P28825	Meprin A subunit alpha	0.0205	0.326	0.000155	0.424	YES	YES
Q3UNX5	Acyl-coenzyme A synthetase ACSM3, mitochondrial	0.0289	0.431	0.00257	0.203	NO	YES
Q9DCC4	Pyrroline-5-carboxylate reductase 3	0.0356	0.537	0.0204	0.287	NO	YES

**Table 5:** Biological function of the most significant consistently changed glomerular proteins associated with early and late DKD. The biological function of the most significant consistently changed glomerular proteins associated with early and late DKD. The upregulated (green color) and downregulated proteins (red color) in Ins2Akita compared to WT controls are shown below.

GO Term	Group *p* Value Corrected with BH	Group Genes
Cellular amino acid catabolic process	2.51 × 10^−6^	Aass, Amt, Gcat, Gldc, Gls
Alpha-amino acid catabolic process	2.83 × 10^−6^	Aass, Amt, Gcat, Gldc, Gls
Response to mercury ion	0.0004	Aqp1, Cds2, Dysf, Fn1, Krt10
Cellular response to osmotic stress	0.00208	Aqp1, Cds2, Dysf
Fatty acid catabolic process	2.36 × 10^−7^	Acox1, Ces1f, Crot, Ehhadh, Pipox, Pycrl
Isoprenoid catabolic process	0.0218	Amacr
Ether lipid biosynthetic process	0.0275	Agps
Trans-2-enoyl-CoA reductase (NADPH) activity	0.029	Pecr

## Data Availability

All data are available in the main text and the Supplementary Files.

## Supplementary Materials

The following supporting information can be downloaded at: https://www.mdpi.com/article/10.3390/biomedicines10020216/s1, Figure S1. Kinetics of weight, glycemia and ACR in Ins2Akita and db/db mice. Blood and urine of controls (wt and db/dm, respectively) and Ins2Akita (A) and db/db (B) mice were collected at different ages and weights and glycemia and ACR were quantified. Values are mean ± SEM. (C) Glomerular damage and fibrosis in db/db mice. Glomerular injury (PAS staining) (A) and interstitial fibrosis (Masson trichrome staining) (B) were quantified in kidney slices from 4-month-old non-diabetic db/m (*n* = 8) and diabetic db/db mice (*n* = 6). Values are mean ± SEM and a Mann–Whitney test was used for comparison. *** *p* < 0.001; ** *p* < 0.01.; Figure S2. Correlation analysis of the protein abundance between our dataset and the study of Waanders et al. 2009 [37] (PMID 19846766). A correlation factor of R = 0.7 for the wild-type mice and R = 0.7 for the Ins2Akita mice was observed; Table S1. Summary of DKD datasets extracted from Nephroseq database; Table S2. The main clinicopathological parameters of the 16 human cases of IHC analysis; Table S3. The full list of all proteomics data of each sample of wild-type and Ins2Akita mice at 2 and 4 months old, respectively; Table S4. The full list of identified proteins using a 55% frequency threshold of wild-type and Ins2Akita mice at 2 and 4 months old, respectively, including the fold change (ratio) and the statistics; Table S5. The biological process of the upregulated and downregulated proteins comparing Ins2akita vs. WT at 2 months old and 4 months old, respectively; Table S6. Statistically significant changed proteins comparing WT at 4 months old vs. WT at 2 months old; Table S7. List of the glomerular proteins consistently changed in early and late DKD versus controls which are related to diabetes and/or have attributed kidney expression and/or function; Table S8, The full list of all proteomics data of each sample of wild-type and db/db mice at 6 months old; Table S9. A comprehensive list of the identified proteins using a 60% frequency threshold of wild-type and db/db mice at 6 months old including the fold change (ratio) and the statistics; Table S10. Cross-omics validation of deregulated consistently changed mitochondrial and peroxisomal glomerular proteins in early and late DKD. The upregulated proteins are presented in green and the downregulated in red color; Table S11. Cross-omics validation of additional peroxisomal deregulated proteins detected in the cortex collected from db/db mice with late DKD. The downregulated proteins are presented in red color.

## References

[1] International Diabetes Federation (2019). IDF Diabetes Atlas.

[2] Zheng Y., Ley S.H., Hu F.B. (2018). Global aetiology and epidemiology of type 2 diabetes mellitus and its complications. Nat. Rev. Endocrinol..

[3] Amatruda M., Gembillo G., Giuffrida A.E., Santoro D., Conti G. (2021). The Aggressive Diabetic Kidney Disease in Youth-Onset Type 2 Diabetes: Pathogenetic Mechanisms and Potential Therapies. Medicina.

[4] Kainz A., Hronsky M., Stel V.S., Jager K.J., Geroldinger A., Dunkler D., Heinze G., Tripepi G., Oberbauer R. (2015). Prediction of prevalence of chronic kidney disease in diabetic patients in countries of the European Union up to 2025. Nephrol. Dial. Transplant..

[5] Gembillo G., Ingrasciotta Y., Crisafulli S., Luxi N., Siligato R., Santoro D., Trifirò G. (2021). Kidney Disease in Diabetic Patients: From Pathophysiology to Pharmacological Aspects with a Focus on Therapeutic Inertia. Int. J. Mol. Sci..

[6] Narres M., Claessen H., Droste S., Kvitkina T., Koch M., Kuss O., Icks A. (2016). The incidence of end-stage renal disease in the diabetic (compared to the non-diabetic) population: A sys-tematic review. PLoS ONE.

[7] Halimi J.-M. (2012). The emerging concept of chronic kidney disease without clinical proteinuria in diabetic patients. Diabetes Metab..

[8] Santoro D., Torreggiani M., Pellicanò V., Cernaro V., Messina R., Longhitano E., Siligato R., Gembillo G., Esposito C., Piccoli G. (2021). Kidney Biopsy in Type 2 Diabetic Patients: Critical Reflections on Present Indications and Diagnostic Alternatives. Int. J. Mol. Sci..

[9] Badal S.S., Danesh F.R. (2014). New Insights into Molecular Mechanisms of Diabetic Kidney Disease. Am. J. Kidney Dis..

[10] Hathaway C.K., Gasim A.M.H., Grant R., Chang A.S., Kim H.-S., Madden V.J., Bagnell C.R., Jennette J.C., Smithies O., Kakoki M. (2015). Low TGFβ1 expression prevents and high expression exacerbates diabetic nephropathy in mice. Proc. Natl. Acad. Sci. USA.

[11] Lopez-Sanz L., Bernal S., Recio C., Lazaro I., Oguiza A., Melgar A., Jimenez-Castilla L., Egido J., Gomez-Guerrero C. (2018). SOCS1-targeted therapy ameliorates renal and vascular oxidative stress in diabetes via STAT1 and PI3K inhibition. Lab. Investig..

[12] Jing Z., Hu L., Su Y., Ying G., Ma C., Wei J. (2021). Potential signaling pathway through which Notch regulates oxidative damage and apoptosis in renal tubular epithelial cells induced by high glucose. J. Recept. Signal Transduct..

[13] Srivastava S.P., Zhou H., Setia O., Liu B., Kanasaki K., Koya D., Dardik A., Fernandez-Hernando C., Goodwin J. (2021). Loss of endothelial glucocorticoid receptor accelerates diabetic nephropathy. Nat. Commun..

[14] Srivastava S.P., Li J., Takagaki Y., Kitada M., Goodwin J.E., Kanasaki K., Koya D. (2021). Endothelial SIRT3 regulates myofibroblast metabolic shifts in diabetic kidneys. iScience.

[15] Klein J., Caubet C., Camus M., Makridakis M., Denis C., Gilet M., Feuillet G., Rascalou S., Neau E., Garrigues L. (2020). Connectivity mapping of glomerular proteins identifies dimethylaminoparthenolide as a new inhibitor of diabetic kidney disease. Sci. Rep..

[16] Granata S., Zaza G., Simone S., Villani G., Latorre D., Pontrelli P., Carella M., Schena F.P., Grandaliano G., Pertosa G. (2009). Mitochondrial dysregulation and oxidative stress in patients with chronic kidney disease. BMC Genom..

[17] Daenen K., Andries A., Mekahli D., Van Schepdael A., Jouret F., Bammens B. (2019). Oxidative stress in chronic kidney disease. Pediatr. Nephrol..

[18] Youssef N., Noureldein M., Njeim R., Ghadieh H.E., Harb F., Azar S.T., Fares N., Eid A.A. (2021). Reno-Protective Effect of GLP-1 Receptor Agonists in Type1 Diabetes: Dual Action on TRPC6 and NADPH Oxidases. Biomed..

[19] Tagawa A., Yasuda M., Kume S., Yamahara K., Nakazawa J., Chin-Kanasaki M., Araki H., Araki S.-I., Koya D., Asanuma K. (2015). Impaired Podocyte Autophagy Exacerbates Proteinuria in Diabetic Nephropathy. Diabetes.

[20] Yoshibayashi M., Kume S., Yasuda-Yamahara M., Yamahara K., Takeda N., Osawa N., Chin-Kanasaki M., Nakae Y., Yokoi H., Mukoyama M. (2020). Protective role of podocyte autophagy against glomerular endothelial dysfunction in diabetes. Biochem. Biophys. Res. Commun..

[21] Oberg B.P., McMenamin E., Lucas F.L., McMonagle E., Morrow J., Ikizler T.A., Himmelfarb J. (2004). Increased prevalence of oxidant stress and inflammation in patients with moderate to severe chronic kidney disease. Kidney Int..

[22] Kang H.M., Ahn S.H., Choi P., Ko Y.-A., Han S.H., Chinga F., Park A.S.D., Tao J., Sharma K., Pullman J. (2015). Defective fatty acid oxidation in renal tubular epithelial cells has a key role in kidney fibrosis development. Nat. Med..

[23] Duni A., Liakopoulos V., Roumeliotis S., Peschos D., Dounousi E. (2019). Oxidative Stress in the Pathogenesis and Evolution of Chronic Kidney Disease: Untangling Ariadne’s Thread. Int. J. Mol. Sci..

[24] Izquierdo-Lahuerta A., Martínez-García C., Medina-Gómez G. (2016). Lipotoxicity as a trigger factor of renal disease. J. Nephrol..

[25] Herman-Edelstein M., Scherzer P., Tobar A., Levi M., Gafter U. (2014). Altered renal lipid metabolism and renal lipid accumulation in human diabetic nephropathy. J. Lipid Res..

[26] Townsend R.R., Guarnieri P., Argyropoulos C., Blady S., Boustany-Kari C.M., Devalaraja-Narashimha K., Morton L., Mottl A.K., Patel U., Palmer M. (2020). Rationale and design of the Transformative Research in Diabetic Nephropathy (TRIDENT) Study. Kidney Int..

[27] Heerspink H.J.L., Jongs N., Chertow G.M., Langkilde A.M., McMurray J.J.V., Correa-Rotter R., Rossing P., Sjöström C.D., Stefansson B.V., Toto R.D. (2021). Effect of dapagliflozin on the rate of decline in kidney function in patients with chronic kidney disease with and without type 2 diabetes: A prespecified analysis from the DAPA-CKD trial. Lancet Diabetes Endocrinol..

[28] Perkovic V., Jardine M.J., Neal B., Bompoint S., Heerspink H.J.L., Charytan D.M., Edwards R., Agarwal R., Bakris G., Bull S. (2019). Canagliflozin and Renal Outcomes in Type 2 Diabetes and Nephropathy. N. Engl. J. Med..

[29] Delanaye P., Wissing K.M., Scheen A.J. (2021). Sodium–glucose cotransporter 2 inhibitors: Renal outcomes according to baseline albuminuria. Clin. Kidney J..

[30] Giugliano D., Maiorino M.I., Bellastella G., Longo M., Chiodini P., Esposito K. (2019). GLP-1 receptor agonists for prevention of cardiorenal outcomes in type 2 diabetes: An updated meta-analysis including the REWIND and PIONEER 6 trials. Diabetes Obes. Metab..

[31] Kanasaki K., Shi S., Kanasaki M., He J., Nagai T., Nakamura Y., Ishigaki Y., Kitada M., Srivastava S.P., Koya D. (2014). Linagliptin-Mediated DPP-4 Inhibition Ameliorates Kidney Fibrosis in Streptozotocin-Induced Diabetic Mice by Inhibiting Endothelial-to-Mesenchymal Transition in a Therapeutic Regimen. Diabetes.

[32] Wang J., Xiang H., Lu Y., Wu T., Ji G. (2021). New progress in drugs treatment of diabetic kidney disease. Biomed. Pharmacother..

[33] Barrera-Chimal J., Lima-Posada I., Bakris G.L., Jaisser F. (2021). Mineralocorticoid receptor antagonists in diabetic kidney disease—Mechanistic and therapeutic effects. Nat. Rev. Nephrol..

[34] Kitada M., Ogura Y., Koya D. (2016). Rodent models of diabetic nephropathy: Their utility and limitations. Int. J. Nephrol. Renov. Dis..

[35] Susztak K., Raff A.C., Schiffer M., Böttinger E.P. (2006). Glucose-induced reactive oxygen species cause apoptosis of podocytes and podocyte depletion at the onset of diabetic nephropathy. Diabetes.

[36] Katsuda Y., Ohta T., Shinohara M., Bin T., Yamada T. (2013). Diabetic mouse models. Open J. Anim. Sci..

[37] Waanders L.F., Chwalek K., Monetti M., Kumar C., Lammert E., Mann M. (2009). Quantitative proteomic analysis of single pancreatic islets. Proc. Natl. Acad. Sci. USA.

[38] Woroniecka K.I., Park A.S.D., Mohtat D., Thomas D.B., Pullman J.M., Susztak K. (2011). Transcriptome Analysis of Human Diabetic Kidney Disease. Diabetes.

[39] Schmid H., Boucherot A., Yasuda Y., Henger A., Brunner B., Eichinger F., Nitsche A., Kiss E., Bleich M., Gröne H.-J. (2006). Modular Activation of Nuclear Factor-κB Transcriptional Programs in Human Diabetic Nephropathy. Diabetes.

[40] Hodgin J.B., Nair V., Zhang H., Randolph A., Harris R.C., Nelson R.G., Weil E.J., Cavalcoli J.D., Patel J.M., Brosius F.C. (2013). Identification of Cross-Species Shared Transcriptional Networks of Diabetic Nephropathy in Human and Mouse Glomeruli. Diabetes.

[41] Wilson P.C., Wu H., Kirita Y., Uchimura K., Ledru N., Rennke H.G., Welling P.A., Waikar S.S., Humphreys B.D. (2019). The single-cell transcriptomic landscape of early human diabetic nephropathy. Proc. Natl. Acad. Sci. USA.

[42] Amiri F.S. (2019). Intracellular organelles in health and kidney disease. Néphrologie Thérapeutique.

[43] Lukey M.J., Wilson K.F., A Cerione R. (2013). Therapeutic strategies impacting cancer cell glutamine metabolism. Futur. Med. Chem..

[44] Cheng S., Rhee E.P., Larson M., Lewis G.D., McCabe E.L., Shen D., Palma M.J., Roberts L., Dejam A., Souza A.L. (2012). Metabolite Profiling Identifies Pathways Associated With Metabolic Risk in Humans. Circulation.

[45] Bao Y., Zhao T., Wang X., Qiu Y., Su M., Jia W., Jia W. (2009). Metabonomic Variations in the Drug-Treated Type 2 Diabetes Mellitus Patients and Healthy Volunteers. J. Proteome Res..

[46] Newgard C.B. (2012). Interplay between Lipids and Branched-Chain Amino Acids in Development of Insulin Resistance. Cell Metab..

[47] Menge B.A., Schrader H., Ritter P.R., Ellrichmann M., Uhl W., Schmidt W.E., Meier J.J. (2010). Selective amino acid deficiency in patients with impaired glucose tolerance and type 2 diabetes. Regul. Pept..

[48] Ferrannini E., Natali A., Camastra S., Nannipieri M., Mari A., Adam K.-P., Milburn M.V., Kastenmüller G., Adamski J., Tuomi T. (2013). Early Metabolic Markers of the Development of Dysglycemia and Type 2 Diabetes and Their Physiological Significance. Diabetes.

[49] Simmons R.M., McKnight S.M., Edwards A.K., Wu G., Satterfield M.C. (2020). Obesity increases hepatic glycine dehydrogenase and aminomethyltransferase expression while dietary glycine supplementation reduces white adipose tissue in Zucker diabetic fatty rats. Amino Acids.

[50] Gar C., Rottenkolber M., Prehn C., Adamski J., Seissler J., Lechner A. (2017). Serum and plasma amino acids as markers of prediabetes, insulin resistance, and incident diabetes. Crit. Rev. Clin. Lab. Sci..

[51] Alves A., Bassot A., Bulteau A.-L., Pirola L., Morio B. (2019). Glycine Metabolism and Its Alterations in Obesity and Metabolic Diseases. Nutrients.

[52] Palmer N.D., Stevens R.D., Antinozzi P.A., Anderson A., Bergman R.N., Wagenknecht L.E., Newgard C.B., Bowden D.W. (2015). Metabolomic Profile Associated With Insulin Resistance and Conversion to Diabetes in the Insulin Resistance Atherosclerosis Study. J. Clin. Endocrinol. Metab..

[53] Sekhar R.V., McKay S.V., Patel S.G., Guthikonda A.P., Reddy V.T., Balasubramanyam A., Jahoor F. (2010). Glutathione Synthesis Is Diminished in Patients with Uncontrolled Diabetes and Restored by Dietary Supplementation with Cysteine and Glycine. Diabetes Care.

[54] Yamakado M., Nagao K., Imaizumi A., Tani M., Toda A., Tanaka T., Jinzu H., Miyano H., Yamamoto H., Daimon T. (2015). Plasma Free Amino Acid Profiles Predict Four-Year Risk of Developing Diabetes, Metabolic Syndrome, Dyslipidemia and Hypertension in Japanese Population. Sci. Rep..

[55] Lanza I., Zhang S., Ward L.E., Karakelides H., Raftery D., Nair K.S. (2010). Quantitative Metabolomics by 1H-NMR and LC-MS/MS Confirms Altered Metabolic Pathways in Diabetes. PLoS ONE.

[56] Neinast M., Murashige D., Arany Z. (2019). Branched chain amino acids. Annu. Rev. Physiol..

[57] Cai F., Zhou X., Jia Y., Yao W., Lv J., Liu G., Yang L. (2020). Identification of Key Genes of Human Advanced Diabetic Nephropathy Independent of Proteinuria by Transcriptome Analysis. BioMed Res. Int..

[58] Van Veldhoven P.P. (2010). Biochemistry and genetics of inherited disorders of peroxisomal fatty acid metabolism. J. Lipid Res..

[59] Wanders R., Ferdinandusse S., Brites P., Kemp S. (2010). Peroxisomes, lipid metabolism and lipotoxicity. Biochim. Biophys. Acta (BBA) Mol. Cell Biol. Lipids.

[60] Silcox A., Burdett K., Connock M.J. (1983). Reduced levels of peroxisomal enzymes in the kidney of the genetically obese (ob/ob) mouse. Contrast with liver. Biochem. Int..

[61] Proctor G., Jiang T., Iwahashi M., Wang Z., Li J., Levi M. (2006). Regulation of Renal Fatty Acid and Cholesterol Metabolism, Inflammation, and Fibrosis in Akita and OVE26 Mice with Type 1 Diabetes. Diabetes.

[62] Vasko R. (2016). Peroxisomes and Kidney Injury. Antioxidants Redox Signal..

[63] Houten S.M., Denis S., Argmann C.A., Jia Y., Ferdinandusse S., Reddy J.K., Wanders R.J.A. (2012). Peroxisomal L-bifunctional enzyme (Ehhadh) is essential for the production of medium-chain dicarboxylic acids. J. Lipid Res..

[64] Violante S., Achetib N., van Roermund C.W.T., Hagen J., Dodatko T., Vaz F.M., Waterham H.R., Chen H., Baes M., Yu C. (2019). Peroxisomes can oxidize medium- and long-chain fatty acids through a pathway involving ABCD3 and HSD17B4. FASEB J..

[65] Kumar A., Shiloach J., Betenbaugh M.J., Gallagher E.J. (2015). The beta-3 adrenergic agonist (CL-316,243) restores the expression of down-regulated fatty acid oxidation genes in type 2 diabetic mice. Nutr. Metab..

[66] Gholaminejad A., Fathalipour M., Roointan A. (2021). Comprehensive analysis of diabetic nephropathy expression profile based on weighted gene co-expression network analysis algorithm. BMC Nephrol..

[67] Brites P., Waterham H.R., Wanders R.J. (2004). Functions and biosynthesis of plasmalogens in health and disease. Biochim. Biophys. Acta (BBA) Mol. Cell Biol. Lipids.

[68] Wallner S., Schmitz G. (2011). Plasmalogens the neglected regulatory and scavenging lipid species. Chem. Phys. Lipids.

[69] Oresic M., Simell S., Sysi-Aho M., Nanto-Salonen K., Seppanen-Laakso T., Parikka V., Katajamaa M., Hekkala A., Mattila I., Keskinen P. (2008). Dysregulation of lipid and amino acid metabolism precedes islet autoimmunity in children who later progress to type 1 diabetes. J. Exp. Med..

[70] Lindfors E., Gopalacharyulu P.V., Halperin E., Oresic M. (2009). Detection of Molecular Paths Associated with Insulitis and Type 1 Diabetes in Non-Obese Diabetic Mouse. PLoS ONE.

[71] Colas R., Pruneta-Deloche V., Guichardant M., Luquain-Costaz C., Cugnet-Anceau C., Moret M., Vidal H., Moulin P., Lagarde M., Calzada C. (2010). Increased Lipid Peroxidation in LDL from Type-2 Diabetic Patients. Lipids.

[72] Natarajan S.K., Muthukrishnan E., Khalimonchuk O., Mott J., Becker D.F. (2017). Evidence for Pipecolate Oxidase in Mediating Protection Against Hydrogen Peroxide Stress. J. Cell. Biochem..

[73] Van Schaftingen E., Rzem R., Marbaix A., Collard F., Veiga-Da-Cunha M., Linster C.L. (2013). Metabolite proofreading, a neglected aspect of intermediary metabolism. J. Inherit. Metab. Dis..

[74] Dahabieh M.S., Di Pietro E., Jangal M., Goncalves C., Witcher M., Braverman N.E., del Rincón S.V. (2018). Peroxisomes and cancer: The role of a metabolic specialist in a disease of aberrant metabolism. Biochim. Biophys. Acta.

[75] Fall T., Salihovic S., Brandmaier S., Nowak C., Ganna A., Gustafsson S., Broeckling C., Prenni J., Kastenmüller G., Peters A. (2016). Non-targeted metabolomics combined with genetic analyses identifies bile acid synthesis and phospholipid metabolism as being associated with incident type 2 diabetes. Diabetologia.

[76] Shumar S.A., Kerr E.W., Geldenhuys W.J., Montgomery G.E., Fagone P., Thirawatananond P., Saavedra H., Gabelli S., Leonardi R. (2018). Nudt19 is a renal CoA diphosphohydrolase with biochemical and regulatory properties that are distinct from the hepatic Nudt7 isoform. J. Biol. Chem..

[77] Hunt M.C., Tillander V., Alexson S.E. (2014). Regulation of peroxisomal lipid metabolism: The role of acyl-CoA and coenzyme A metabolizing enzymes. Biochimie.

[78] Ebaid H., Bashandy S.A.E., Abdel-Mageed A.M., Al-Tamimi J., Hassan I., Alhazza I.M. (2020). Folic acid and melatonin mitigate diabetic nephropathy in rats via inhibition of oxidative stress. Nutr. Metab..

[79] Hwang I., Lee J., Huh J.Y., Park J., Lee H.B., Ho Y.-S., Ha H. (2012). Catalase Deficiency Accelerates Diabetic Renal Injury Through Peroxisomal Dysfunction. Diabetes.

[80] Oguro A., Fujita N., Imaoka S. (2009). Regulation of Soluble Epoxide Hydrolase (sEH) in Mice with Diabetes: High Glucose Suppresses sEH Expression. Drug Metab. Pharmacokinet..

[81] Jung O., Jansen F., Mieth A., Barbosa-Sicard E., Pliquett R.U., Babelova A., Morisseau C., Hwang S.H., Tsai C., Hammock B.D. (2010). Inhibition of the Soluble Epoxide Hydrolase Promotes Albuminuria in Mice with Progressive Renal Disease. PLoS ONE.

[82] Pollegioni L., Sacchi S., Murtas G. (2018). Human D-Amino Acid Oxidase: Structure, Function, and Regulation. Front. Mol. Biosci..

[83] Satav J.G., Dave K.R., Katyare S.S. (2000). Influence of Insulin Status on Extra-Mitochondrial Oxygen Metabolism in the Rat. Horm. Metab. Res..

[84] Latosinska A., Davalieva K., Makridakis M., Mullen W., Schanstra J.P., Vlahou A., Mischak H., Frantzi M. (2020). Molecular Changes in Tissue Proteome During Prostate Cancer Development: Proof-of-Principle Investigation. Diagnostics.

[85] Makridakis M., Vlahou A. (2018). GeLC-MS: A sample preparation method for proteomics analysis of minimal amount of tissue. Methods Mol. Biol..

[86] Latosinska A., Vougas K., Makridakis M., Klein J., Mullen W., Abbas M., Stravodimos K., Katafigiotis I., Merseburger A.S., Zoidakis J. (2015). Comparative Analysis of Label-Free and 8-Plex iTRAQ Approach for Quantitative Tissue Proteomic Analysis. PLoS ONE.

[87] Tervaert T.W.C., Mooyaart A.L., Amann K., Cohen A.H., Cook H.T., Drachenberg C.B., Ferrario F., Fogo A.B., Haas M., de Heer E. (2010). Pathologic Classification of Diabetic Nephropathy. J. Am. Soc. Nephrol..

[88] eGFR Calculator National Kidney Foundation. https://www.kidney.org/professionals/kdoqi/gfr_calculator.

[89] Stevens P.E., Levin A. (2013). Kidney disease: Improving global outcomes chronic kidney disease guideline development work group members. Evaluation and management of chronic kidney disease: Synopsis of the kidney disease: Improving global outcomes 2012 clinical practice guideline. Ann. Intern. Med..

