# Supplementary material for: Proteomic Analysis of Mouse Kidney Tissue Associates Peroxisomal Dysfunction with Early Diabetic Kidney Disease
Source: Biomedicines. 2022 Jan 20;10(2):216. doi: 10.3390/biomedicines10020216 (PMC8869654; doi:10.3390/biomedicines10020216)
Supplement: Supplementary file 1 [file biomedicines-10-00216-s001.zip › Tables S10 and S11.pdf]

Table S10

Protein	Transcriptomics expression (Nephroseq; in DKD vs. controls) (ref)	Single-cell human kidney transcriptomics expression (Wilson et al. 41)	Protein expression or metabolites (ref)
GLS	increase (38,39)	increase (tubuli)	No proteomics data only on metabolites (43-48) reduced levels of glutamine and/or elevated levels of glutamate in diabetes
GLDC	increase (40 db/db mouse glomeruli)	increase (tubuli)	No proteomics data only on metabolites (48-52) reduced levels of glycine in diabetes
AMT	decrease (38)	not detected	No proteomics data only on metabolites (48-52) reduced levels of glycine in diabetes
ACOX1	no data	decrease (tubuli)	decrease (60,61)
CROT	decrease (40 db/db mouse glomeruli)	decrease (glomeruli)	decrease (60) ob/ob
EHHADH	decrease (39, 40 db/db mouse glomeruli)	decrease (glomeruli)	not detected
AGPS	decrease (40 db/db mouse glomeruli)	decrease (glomeruli)	No proteomics data only on metabolites (68-70) low plasmalogens in diabetes
PIPOX	decrease (39)	not detected	No proteomics data only on metabolites (22, 75) elevated pipecolate in diabetes
AMACR	decrease (39)	decrease (glomeruli)	No proteomics data only on metabolites (75) reduced levels of bile acid synthesis in diabetes
NUDT19	decrease (40 db/db mouse glomeruli)	not detected	not detected
PECR	decrease (40 db/db mouse glomeruli)	not detected	not detected

Table S11

Protein	Transcriptomics expression (Nephroseq; in DKD vs. controls) (ref)	Single-cell human kidney transcriptomics expression (Wilson et al. 41)	Protein expression (ref)
**CAT**	decrease (40 db/db mouse glomeruli)	decrease (tubuli)	decrease (78) STZ mice
**EPHX2**	no data	decrease (tubuli)	not detected
**DAO**	decrease (38,39)	decrease (glomeruli and tubuli)	decrease (83)
