# Supplementary material for: Proteomic Analysis of Mouse Kidney Tissue Associates Peroxisomal Dysfunction with Early Diabetic Kidney Disease
Source: Biomedicines. 2022 Jan 20;10(2):216. doi: 10.3390/biomedicines10020216 (PMC8869654; doi:10.3390/biomedicines10020216)
Supplement: Supplementary file 1 [file biomedicines-10-00216-s001.zip › TableS2.pdf]

Table S2

Case No	Gender	Age	DM history	DKD Class	IFTA score	Inflammation score	Hyalinosis score	Art/sclerosis score	Total score	CKD Stage	SCr (mg/dL)	Albuminuria (g/24h)	eGFR ml/min/1,73 m^2
1	M	51	DM type II	**I**	1	0	2	1	4	**G1A3**	0.9	0.5	99
2	M	55	DM type II	**IIa**	2	1	2	1	6	**G1A3**	0.8	4 – 6	101
3	M	53	DM type II	**IIa**	2	1	2	1	6	**G1A3**	0.95	5.4	91
4	M	50	DM type II	**IIa**	2	1	2	1	6	**G2A3**	1.2	1.8	70
5	M	82	DM type II	**IIb**	3	2	2	2	9	**G2A3**	1	≥3	70
6	M	30	DM type I	**IIb**	3	1	2	1	7	**G3aA3**	1.7	8	53
7	F	35	DM type I	**IIb**	3	1	2	2	8	**G3aA3**	1.3	16.8	53
8	M	73	DM type II	**IIb**	2	1	2	2	7	**G3bA3**	1.6	≥3	42
9	M	58	DM type II	**III**	3	1	2	2	8	**G3bA3**	2	3	36
10	M	80	DM type II	**III**	3	1	2	2	8	**G3bA3**	1.9	≥3	33
11	M	69	DM type II	**III**	2	1	2	0	5	**G3bA3**	2	4	33
12	M	58	DM type II	**III**	3	1	2	2	8	**G3bA3**	2.2	≥3	32
13	M	65	DM type II	**IV**	3	1	2	2	8	**G4A3**	2.9	2	22
14	F	62	DM type I	**IV**	3	1	2	2	8	**G4A3**	2.5	0.6	20
15	M	50	DM type II	**IV**	3	1	2	2	8	**G5A3**	5.5	≥3	11
16	M	44	DM type I	**IV**	3	1	2	2	8	**G5A3**	7	10	9
