# Supplementary figures and images for: Proteomic Analysis of Mouse Kidney Tissue Associates Peroxisomal Dysfunction with Early Diabetic Kidney Disease
Source: Biomedicines. 2022 Jan 20;10(2):216. doi: 10.3390/biomedicines10020216 (PMC8869654; doi:10.3390/biomedicines10020216)

Figure S1

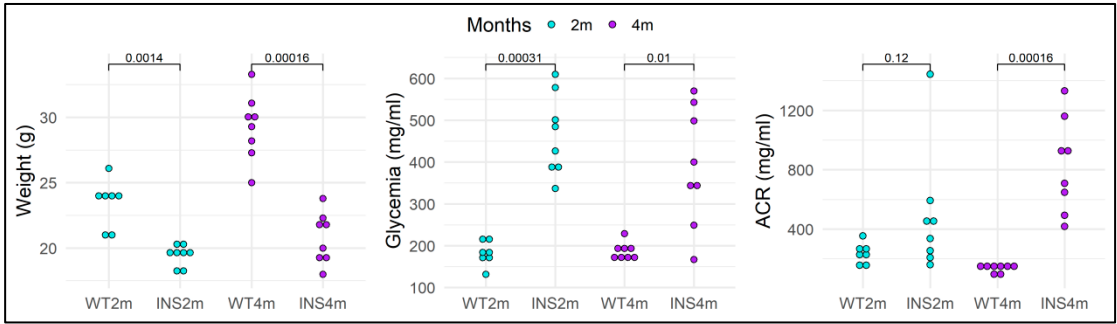

A)

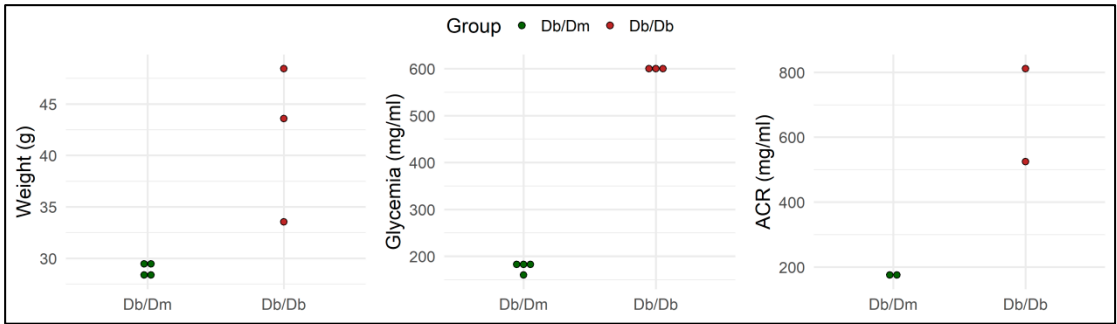

B)

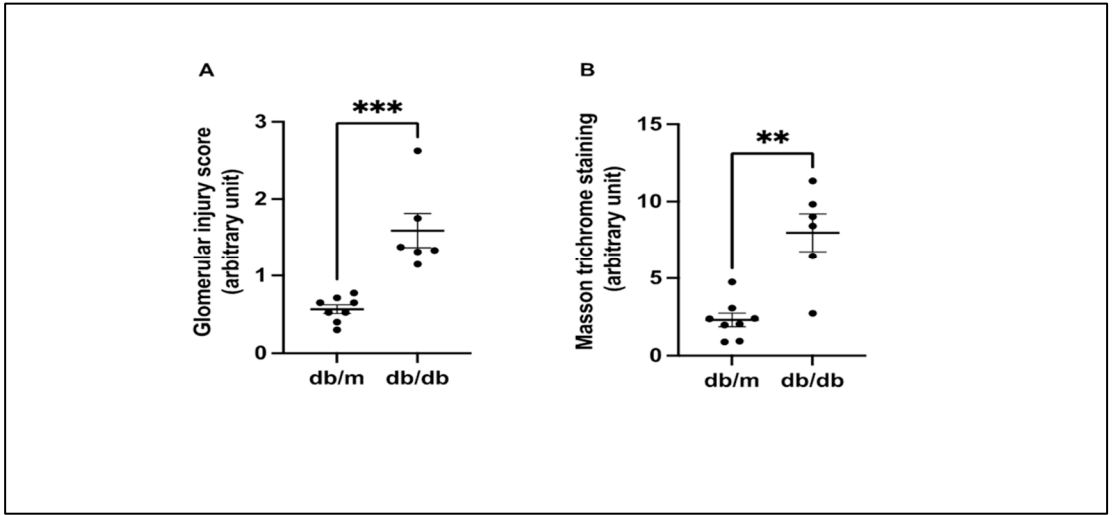

C)

Supplement: Supplementary file 1 [file biomedicines-10-00216-s001.zip › Figure S1.pdf]

Figure S2

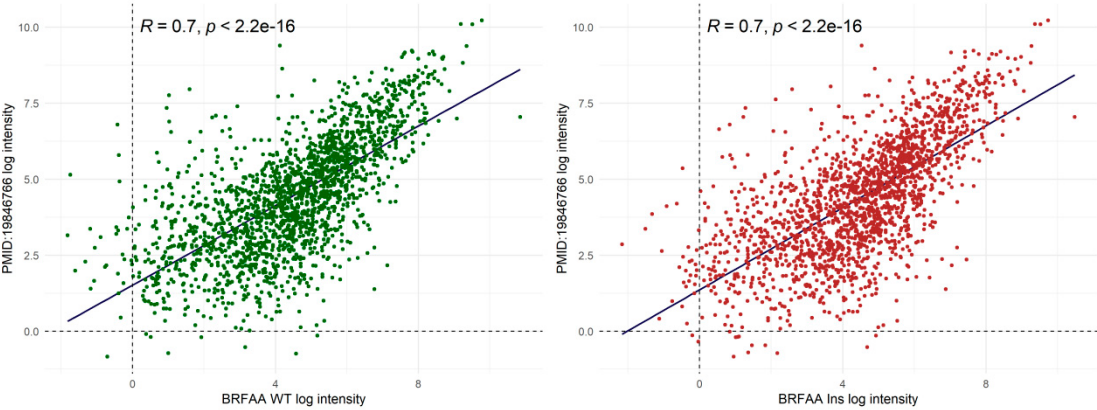

Supplement: Supplementary file 1 [file biomedicines-10-00216-s001.zip › Figure S2.pdf]
